# Ensemble machine learning of factors influencing COVID-19 across US counties

**DOI:** 10.1038/s41598-021-90827-x

**Published:** 2021-06-03

**Authors:** David McCoy, Whitney Mgbara, Nir Horvitz, Wayne M. Getz, Alan Hubbard

**Affiliations:** 1 Division of Environmental Health Sciences, UC Berkeley, Berkeley, CA 94720 USA; 2 Department of Environmental Science, Policy, and Management, UC Berkeley, Berkeley, CA 94720 USA; 3 School of Mathematical Sciences, University of Kwazulu-Natal, Durban, 4000 South Africa; 4 Division Biostatistics, UC Berkeley, Berkeley, CA 94720-3114 USA

**Keywords:** Computational biology and bioinformatics, Data integration, Machine learning, Statistical methods

## Abstract

Severe acute respiratory syndrome coronavirus 2 (SARS-CoV-2) the causal agent for COVID-19, is a communicable disease spread through close contact. It is known to disproportionately impact certain communities due to both biological susceptibility and inequitable exposure. In this study, we investigate the most important health, social, and environmental factors impacting the early phases (before July, 2020) of per capita COVID-19 transmission and per capita all-cause mortality in US counties. We aggregate county-level physical and mental health, environmental pollution, access to health care, demographic characteristics, vulnerable population scores, and other epidemiological data to create a large feature set to analyze per capita COVID-19 outcomes. Because of the high-dimensionality, multicollinearity, and unknown interactions of the data, we use ensemble machine learning and marginal prediction methods to identify the most salient factors associated with several COVID-19 outbreak measure. Our variable importance results show that measures of ethnicity, public transportation and preventable diseases are the strongest predictors for both per capita COVID-19 incidence and mortality. Specifically, the CDC measures for minority populations, CDC measures for limited English, and proportion of Black- and/or African-American individuals in a county were the most important features for per capita COVID-19 cases within a month after the pandemic started in a county and also at the latest date examined. For per capita all-cause mortality at day 100 and total to date, we find that public transportation use and proportion of Black- and/or African-American individuals in a county are the strongest predictors. The methods predict that, keeping all other factors fixed, a 10% increase in public transportation use, all other factors remaining fixed at the observed values, is associated with increases mortality at day 100 of 2012 individuals (95% CI [1972, 2356]) and likewise a 10% increase in the proportion of Black- and/or African-American individuals in a county is associated with increases total deaths at end of study of 2067 (95% CI [1189, 2654]). Using data until the end of study, the same metric suggests ethnicity has double the association as the next most important factors, which are location, disease prevalence, and transit factors. Our findings shed light on societal patterns that have been reported and experienced in the U.S. by using robust methods to understand the features most responsible for transmission and sectors of society most vulnerable to infection and mortality. In particular, our results provide evidence of the disproportionate impact of the COVID-19 pandemic on minority populations. Our results suggest that mitigation measures, including how vaccines are distributed, could have the greatest impact if they are given with priority to the highest risk communities.

## Introduction

### COVID-19 background

Coronavirus disease 2019 (COVID-19), caused by the novel severe acute respiratory syndrome coronavirus 2 (SARS-CoV-2), spread rapidly around the world^1–6^. Within a month of discovering the first cluster of cases in Wuhan, China^1,3,6^, 18 additional countries had reported a case of COVID-19^7^. The World Health Organization declared the resulting outbreaks a Public Health Emergency of International Concern by January 30, 2020 and a pandemic by March 11, 2020^7,8^.

There are a few consistent observations regarding the epidemiology of the COVID-19 pandemic in the US. Most prominent is the relatively high infection and death rates of minority populations, particularly Black- and/or African-Americans^9,10^, a disparity researchers have noted occurred n previous pandemics, such as HIV^11^. This disparity has been observed in both adults and children^12^. There is much previous work on the causes of health disparities among Black- and/or African-Americans, and others have speculated on which of these causes are related to the differential impact of COVID-19^13,14^. Thus, to tease out the impact on vulnerable groups, one needs data on other baseline health factors, such as obesity^15,16^, co-morbidities, age^17,18^ environmental exposures, transportation use and employment factors, including types of occupations^10,19^.

### US health response and forecasting

At the point our analysis was conducted, the US had implemented complex and regionally uneven community-level, non-pharmaceutical interventions, including travel restrictions, social distancing, and stay-at-home orders. Although these interventions have shown to mitigate the community spread in certain communities, the trend did not hold for all communities. Many counties experienced an uptick in cases after an initial decline. There are several reasons why certain communities experienced a growing number of cases, including: (1) lifting shelter-in-place or other social distancing restrictions earlier than advised; (2) lax controls on gatherings that resulted in super-spreader events^20^, and; (3) unknown affects of plausible seasonality that impacts viral transmission^21^. As such, early in the pandemic, complex epidemiological contexts have emerged in US communities. The complexity is a result of dynamic environmental factors constituting social and physical environments for US populations that impact an individual’s risk for contracting COVID-19. Thus, to adequately control the spread of COVID-19, it is important to identify early in the pandemic the most salient social and physical environmental factors within US communities, driving transmission and and effecting susceptibility.

Though models accounting for the specific vulnerabilities of local populations have been proposed, only a few models exist that assess the importance county-level variation of such variables in fueling COVID-19 outbreaks^22,23^. Altieri et al.^22^, for example, use county-level data from similar sources to this paper, to create an ensemble forecasting model, dubbed “Combined Linear and Exponential Predictors” to predict death counts from COVID-19. Their goal is to curate a data repository that can be used to forecast exponential and sub-exponential cases weeks in advance in order to help nonprofit organization disseminate much needed personal protective devices and respirators to areas projected to have higher mortality rates.

In our study, we use overlapping data sources to model cross-sectional COVID-19 outcomes. Our goal, rather than prediction, is to explore the relative importance of different types of social, physical and environmental factors on COVID-19 transmission and mortality. Hospital case and mortality data, and seropositive surveillance studies have shown there are subgroups of the population that are more susceptible to higher cases of morbidity and mortality. These include people older than 65 and and communities affected by racial disparities. We attempt in this paper to expand on previous studies (as of July 2020) of county level variation in COVID-19 (e.g., see^24^) by evaluating additional socio-environmental data to understand if these disparities have direct effects on COVID-19 outcomes or are indirect through additional risk factors, such as diabetes, food security, air pollution or access to health care. Likewise, no study has been carried out to determine which of these factors are most associated with COVID-19 outcomes while still controlling non-parametrically for high dimensional covariates.

Our data set is not comprised of individual-level data but includes a large number of predictor variables with the potential for complicated interactions between sets of risk factors, e.g. the intersection between race/ethnicity, environmental exposures and underlying health on COVID-19 outcomes. To avoid as much as possible model misspecification, we fit a prediction model for each outcome using SuperLearner^25^, an ensemble of machine learning algorithms, which relied on cross-validation across many algorithms to derive an optimal combination of such algorithms. Using this approach, one can derive an improved fit to the data than an arbitrary linear regression model or any one machine learning algorithm. This is important because, in the current study, accurate estimates for our parameters of interest (variable importance and expected COVID-19 cases and/or mortality given a shift in these important variables) is contingent on a good fit to the actual prediction function. Additionally, we are guaranteed to get estimates that are less biased compared to a single pre-specified algorithm because SuperLearner is guaranteed to perform asymptotically as well as the best performing algorithm included in the candidate set of algorithms.

### Added value of this study

We aggregate 5 types of COVID-19 outcomes, (1) day of the first case in a county relative to the first known case reported in the U.S. (Snohomish County, Washington, USA), (2) number of cases 25 days after the initial case in a county, (3) number of all-cause deaths 100 days after the first case in a county, (4) total number of cases in a county to-date after initial case and (5) total number of all-cause deaths in a county to-date after first case in a county. From many sources including the CDC, U.S. Census Bureau, USA facts, google mobility data, and others, we collect a large number of pertinent variables for COVID-19. Using ensemble machine learning (ML) we create models that make no assumptions on the distributions of the data, these models are thereby non-parametric and allow all degrees of interactions and distributions in order to make the best fit.

In each of these models we identify the most important variables in the model by removing the variable and measuring the difference in model risk (model error). Based upon the SuperLearner fits, we make marginal predictions for the number of cases and mortalities from COVID-19 when increasing or decreasing these top variables while controlling for all other factors (i.e., keeping all other predictors fixed at the original values). Confidence intervals (CIs), significance and robustness of findings are measured via bootstrapping the model. Additionally, we investigate the predicted number of cases and mortalities from our model when controlling for only variables outside the target variable category (i.e ethnicity, public transportation subcategories) and univariate predictions (not controlling for other variables). Given this approach, our contributions are, rather than predictive forecasting the number of cases, an approach to measure the relative importance of risk factors for COVID-19 from a county-level perspective.

There are many known risk factors measured from case hospitalization data, including diabetes and heart disease. As such, we hypothesize these factors will show high variable importance, specifically for case mortality. Additionally, we hypothesize that environmental dynamics, which increase exposure time to the virus (i.e., number of occupations in a county, public transit use), will also be strongly associated with COVID-19 cases early in the pandemic. When such factors are identified, this information could also be used to update or improve the public health response to specifically target factors related to high case counts in order to further mitigate new cases or prevent a resurgence of cases. We estimate variable importance of the high dimensional assembled county features without constraints (which induces bias), using a combination of machine learning and intuitive substitution estimators. Lastly, we make both the data and methods used in this paper accessible to others, thereby providing open source access and enhancing the utility of our results. All code, data, and county variables used are available and outcome data are updated daily on GitHub (https://github.com/blind-contours/Getz_Hubbard_Covid_Ensemble_ML_Public.git).

**Figure 1:**
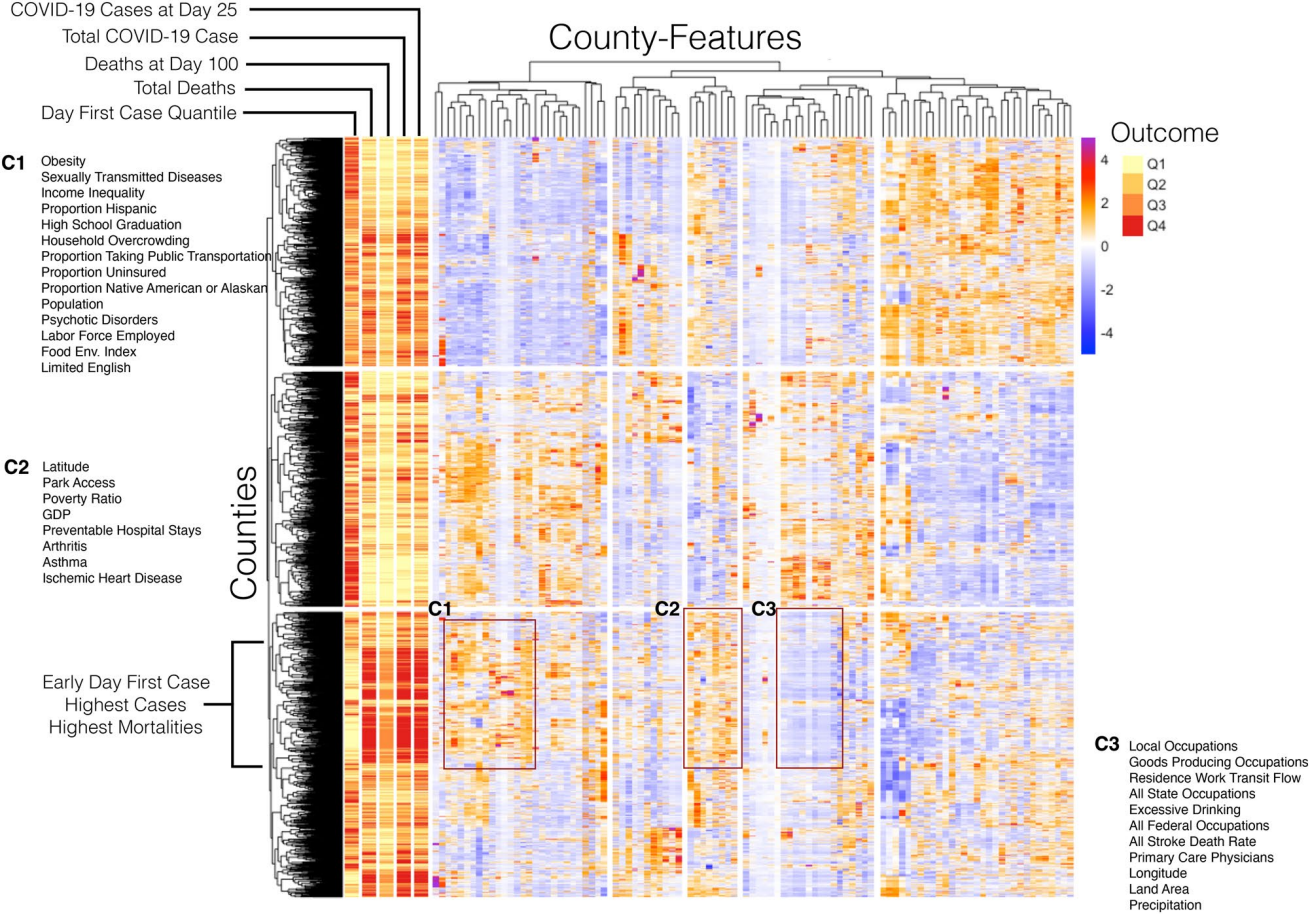
COVID-19 heatmap visualization of the distribution of county-level data. The rows represent counties clustered by the dendrogram and the columns are features of the counties which are also clustered by similarity. Red coloration indicates higher values (up to a z-score of 4) and blue coloration indicates lower values (− 4). The column bars on the left are outcomes, categorized by quantiles. The sections marked by C1, C2, and C3 show similar high or low features of counties in this region which have early COVID-19 appearance and high transmission and mortalities. Figure was created using pheatmap version 1.0.12^28^.

**Figure 2:**
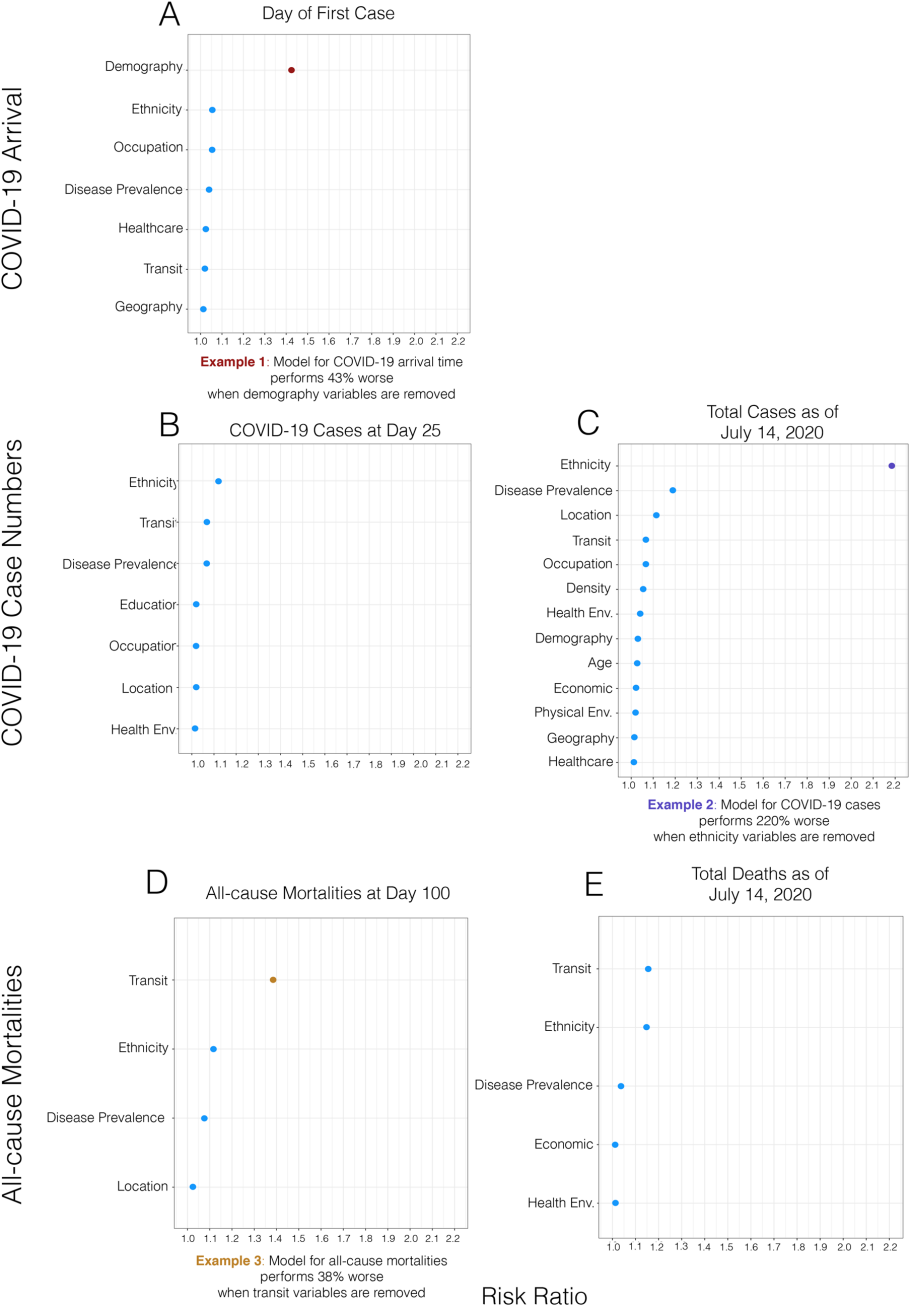
Variable importance as indicated by the relative increase of mean-squared error when the block of variables is permuted.

**Table 1:** Number of variables used from respective sources with some examples given, complete list with distributions given in supplementary material.

Source	N Var.	Var. Examples
USAFacts	6	COVID-19 outcome data, population
Bureau of Economic Analysis (BEA)	1	GDP
5-Year American Community Survey (ACS), 2014–2018	14	County percentages by Sex and Ethnicity, Employment, Household Income, use of Public Transportation
TIGER/Line Geodatabases	7	Latitude, longtitude, land area
TIGER/Line Geodatabases; Federal Aviation Administration (FAA)		Distance to Airports
Interactive Atlas of Heart Disease and Stroke (2014–2016)	4	Number of Hospitals, Stroke, Access to Parks
County Health Rankings and Roadmaps	21	Life Expectancy, Smoking, Obesity,, Food Access, Mental Health, Physicians, Houshold Overcrowding etc.
Centers for Medicare & Medicaid Services (CMS)	15	Druge Abuse, Hypertension, Hyperlipidemia, Osteoporosis, etc.
National Centers for Environmental Information	1	Precipitation
CDC’s Social Vulnerability Index (SVI)	11	Percentile over 65 or under 17, Minority Scores, Limited English, Low Income Housing Estimates, Number Institutionalized
Quarterly Census of Employment and Wages	14	Labor force types, farming/mining, private industry, education/healthcare etc.
MIT election lab	1	Calculated Proportion Voted Republican 2016
Google	6	Google mobility to location type, Residence, Grocery etc.

## Methods

### Data sources

For all outcomes and predictors we compiled publicly available data for US jurisdictions reported at the state-level (e.g. Google Mobility Data) and county-level, excluding Alaska, Hawaii, and Puerto Rico. Our final dataset includes county-level case counts, death counts and a wide variety of county-level demographic, epidemiological, health, and environmental data used as predictors in our analysis. Our analysis was based on the cumulative confirmed cases and deaths of COVID-19 in US counties, starting on January 22 2020 (referred to as Day 1) until July 14 2020 (USA Facts). The sources for our county-level features were: USA Facts, Bureau of Economic Analysis, American Community Survey, Tiger/Line GeoDatabase, CDC Interactive Atlas of Heart Disease and Stroke, County Health Rankings, Centers of Medicare and Medicaid Services, National Centers for Environmental Information, CDC Vulnerability Index, Bureau of Labor Statistics, MIT Election Lab, Google Community Mobility Reports; a total of 12 different sources which were joined on county FIPS codes.

### Outcomes

We use five COVID-19 outcome scenarios: (1) we transformed the case data into day of the first case after the first confirmed case on January 21 2020; (2) we determined the number of cumulative cases on the 25 days after the day of the first case within each county (i.e., day 25 of the outbreak in each county); (3) we used the number of all-cause deaths on day 100 after the day of the first case of the outbreak for each respective county up to July 14 2020; (4) we determine the total number of cases to date after the day of the first case for each county and likewise scenario five is the total number of all-cause mortalities to-date after the day of the first case for each county.

For each outcome variable excluding number of cases at day 25, we divide the counts by population size for each county to create a per capita COVID-19 case or per capita all-cause mortality outcome. Likewise, all predictor variables measured in counts were also standardized by population size. All-cause deaths were used rather than reported fatalities due to COVID-19 for several reasons related to unreliable case data, differences in testing, and co-morbidities between COVID-19 and other fatal acute diseases. By using all-cause deaths measured since day of first case reported in a county, we hope to get a better estimate on the impact of COVID-19 on mortality. As discussed, our predictors cover a wide scope, Table 1 gives a review of the data sources and variables collected from each source. Table S1 in the supplementary section gives more details of this process.

### Predictors

Data on our predictor variables include demographics, health resource availability, health risk factors, social vulnerability, and other COVID-19-related information. The predictor variables used are collected from different sources which have also been used in Altieri et al.^26^ and Killeen et al.^27^. Because the aims of this paper are not purely predictive, but focus on understanding the relative impact each variable has on COVID-19 outcomes, our data curation process is different when compared to these two papers. We aggregate data from different stratified variables to create an overall public transportation use feature. Likewise for social vulnerability scores, we attempt to include core variable that represent a specific type of risk feature. For example, given our interest are variable importance measures and marginal predictions, if we included both the aggregated CDC vulnerability index with many features collected from other sources that are proxy measures for this index (percent non-English speaking, poverty levels etc.) then findings for aggregate vulnerability index would be conservative given these other variables are also included in the model. That being said, given the large overlap and interactions of all variables collected, it it likely that all variable importance estimates are conservative. In our curation process, only unstratified variables are used (not stratified by age or sex), we also create sub-categories for variables (ethnicity, geography, disease prevention, etc.) These sub-categories we use in later analysis to explore possible over correction of the model for a respective target variable.

Briefly, some predictor variables include proportions of individuals by poverty level, gender, age distribution, race distribution, household income, healthcare access, occupation type, and so on, these were collected from USA Facts and the Census Bureau. Airport data, including, distance from county polygon centroid to airports were calculated from the Federal Aviation Administration. The 2020 county health rankings and Center for Medicaid and Medicare services were used to gather information on a range of health data including smoking, diabetes, obesity, air pollution and many other physical and mental health metrics. Precipitation by month was gathered from the National Oceanic and Atmospheric Association. Vulnerability index scores from each theme (described in Table S1) were aggregated from the Center of Disease Control. In total, over 150 predictor variables were gathered before curation. This curated data along with all relevant code, documentation and results are provided on our GitHub page.

### Data cleaning and curation

The data curation process is described in more detail in the supplementary information alongside the data dictionary. All resulting data was numerical (no factor variables). In addition, we screen out any variables with more than 70% missing values. Similarly, we removed variables with close to zero variance. For variables that were nearly perfectly correlated (Pearson correlation = 0.95) we selected one for the analysis. Missing data in this cleaned dataset were imputed with the mean. For Google mobility data, we scraped data from the published mobility trend reports from Feb 16 2020 to March 29 2020. These data represent the general increase or decrease in movement to the respective destination (grocery stores, parks etc.) compared to baseline (pre-pandemic period). To create an aggregate score representing the mobility trend for each movement category for each county, we use the slope from linear regression to measure this trend over time. The slope for each movement category was included in our SuperLearner models.

### Exploratory analysis

To graphically represent how our feature data are related to one another, and likewise how counties are related to one another through these variables, we use unsupervised hierarchical agglomerative clustering of both county features and counties. We present the results of this clustering as a heatmap using the pheatmap package in R^28^ . Our first goal of this method is to understand if there are counties that have a trend for early first case reported, high COVID-19 case rates at day 25, and COVID-19 case rates to-date, as well as high all-cause mortalities at day 100 and to-date. If this trend was seen, we next wanted to investigate what variables were ‘highly expressed’ in these counties. As such, all feature data was z-score standardized. We then took the quantiles of each outcome to create factor dummy variables that can be plotted alongside clustering of counties. Clustering was done for both counties and county features and reordered accordingly using Euclidean distances. As this is an unsupervised approach, outcome data are not included in this machine learning method but are simply plotted alongside the clustering results to visually identify correspondences. Groups of county features that were found to be associated with groups of COVID-19 outcomes are presented.

### Machine learning pipeline

Although our ultimate goal is not to use our final models for forecasting and prediction, one still needs to estimate a regression model in order to determine our measures of variable importance. By estimating this model as accurately as possible, one can better estimate the variable importance measures that rely on the prediction model. As such, instead of choosing one machine learning (ML) algorithm to model county features for each outcome, we used an ensemble approach (SuperLearner) to fit a prediction function for each of our outcomes. The SuperLearner combines the predictive probabilities of COVID-19 outcomes across many ML algorithms. The SuperLearner finds the optimal combination of a collection of algorithms by minimizing the cross-validated risk^25,29^. This method is an improvement over methods using only one ML algorithm because no one algorithm is universally optimal. The SuperLearner has been shown in theory to be at least as good as the best performing algorithm in the ensemble and often times performs considerably better^30^. Given the high-dimensionality and complex relationships of the county data, we chose a wide range of algorithms for the ensemble in order to optimize performance. For COVID-19 cases at day 25 and to-date per capita rates and all-cause mortalities at day 100 and total to-date per capita, we use a large number of linear Gaussian based algorithms including conditional mean (control algorithm) simple generalized linear model, a series of penalized regressions setting alpha at levels to create ridge regression, lasso regression, and elastic net regression^31^. Similarly, we use a number of gradient boosted decision trees that differed in depth^32^. Because these algorithms require hyper-parameter tuning for optimal performance, we create a grid of all possible hyper-parameters and choose algorithms across this grid for inclusion in the ensemble. For example, for xgboost models we create a grid of all combinations for max depth (2,4,6,8,10,12), eta (0.001, 0.01, 0.1, 0.2, 0.3), and nround (20,50) and use models with these hyper-parameters at intervals across the grid. Likewise for decision trees^33^ we select models with max number of trees (10, 50, 100). For elastic net we set alpha to 1 for lasso regression, 0 for ridge and also for alpha set to 0.25, 0.50 and 0.75. Overall, 19 algorithms were used in our Super Leaner library. The same procedure was applied for day-of-first-case in a county relative to-day-of-first case in the U.S. Instead of using Gaussian algorithms, however, custom learners were made for the SuperLearner environment that model Poisson outcome data. The same parameters were chosen for this set of learners to create the Poisson ensemble. To address possible over-fitting and to get cross-validated risks for each algorithm in the ensemble sets, five-fold cross-validation was used for internal SL cross-validation both to build optimal models with each classifier and to determine optimal weighting across classifiers in the ensemble.

### Unpacking the black box

The algorithm used to create our predictor given covariates has desirable optimality properties, being asymptotically guaranteed to have a fit as good as any of the candidate members (the “oracle property”), with no risk of over-fitting. If a library of both smooth (e.g., parametric models) and flexible, non-parametric learners, then one can find it hard to outperform^25,34^. However, the result is a black box that creates predictions as a complex ensemble of different learners, some having their own internal variable selection process and model selection framework. Thus, the resulting black-box needs to be intelligently queried to estimate the independent impact of the various predictors used in the model. We do so in two ways. One, is using a straightforward leave-one-variable out method and re-examining the change in prediction accuracy. However, this provides no information about the direction of the impact, which is why we follow with a query inspired by causal inference methods. In that case, we use the model to forward model situations where we change the distribution of predictors across the counties in sequential fashion and then calculate the marginal predicted counts (so called substitution estimators, or G-computation^35,36^). The combination of these two versions of non-parametric variable importance measures provide both the importance of the variable (or sub-category of variable) to the resulting predictor as well as an intuitive measure of the adjusted association of single variables.

### Variable importance

**Figure 3:**
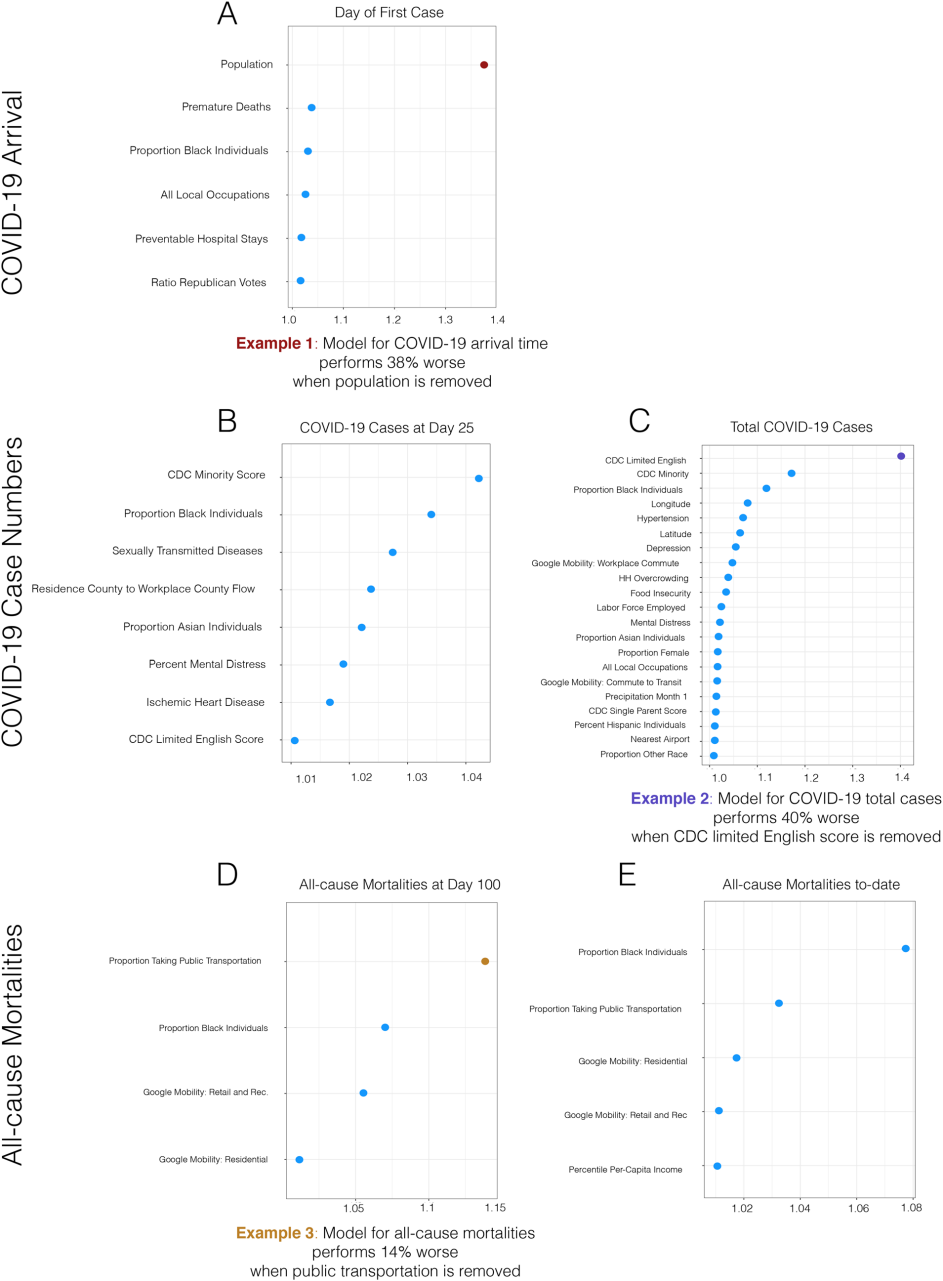
Variable importance as indicated by the relative increase of mean-squared error when a single variable is permuted.

We built SuperLearners from the same county-level data for each of the COVID-19 outcomes. To measure variable importance in each model, we take the fitted model and make predictions using all county features and measure the model risk (average in squared differences in model prediction versus truth, or mean-squared error). We then scramble individual variables and sets (all variable in a category) and re-do the prediction and derive the new MSE. The plots (Figs. 2, 3) show the resulting ranked list of variables (most to least change in the MSE by scrambling). We use a risk-ratio (MSE-ratio) for each variable to measure its relative importance in the model for each outcome. The risk-ratio is the risk in the model without the respective variable (numerator) over the risk when the variable is included in the model (denominator). As such, a risk-ratio of 1.5 indicates that the model MSE rises by 50% when the variable is scrambled while controlling for all other variable affects. We use a similar approach to measure the variable sub-category importance on each outcome. Each variable was given a sub-category (described in supplementary material) resulting in a total of 15 categories. Blocks of variables in each category were scrambled and the model risk-ratio measured to attain information on category importance.

### Marginal predictions

Given that we fit a black-box to derive our prediction models, we have to unpack the black-box to understand what it implies about the adjusted relationship of the important variables to the outcomes. We thus use substitution methods to evaluate the predicted change in the mean if county characteristics are changed. We examine how the mean outcomes would be predicted to change if the inputs of the specific variable of interest are modified, such as reducing a variable in some equivalent way across counties. Other modifications of the inputs could be used to examine these variable importance plots, but we looked at % changes in the variable across counties. Using these models we then make marginal predictions on the predicted number of COVID-19 cases and all-cause mortalities when increasing (or decreasing) the top variables found by the variable importance procedure. For example, if heart disease were to be identified as a significant predictor for COVID-19 mortality, the question our forward modeling approach answers is, “What are the expected number of COVID-19 deaths if we were to reduce the number of people with heart disease by 25% across all counties in the U.S.?”. Similarly, another question is, “What is the trend in expected COVID-19 cases given a incremental decreases in heart disease, say a decrease by 10%, 20% ...90%, is this trend linear or nonlinear?”. And furthermore, “If the trend is linear, what is the average decrease in expected COVID-19 deaths for a 10% reduction in heart disease?”. The non-parametric, forward modeling approach detailed below aims to answer these questions.

Suppose a particular observation $O_i=(W_i,A_i,Y_i)$ in county $i,\ i=1,\ldots ,n$, depends on explanatory variable $A_i$, other adjustment covariates $W_i$, and outcome $Y_i$, all in county *i*. If we wish to generate an estimate that characterizes the association of *Y* across all counties with *A* adjusting for *W*, but does not rely on a linear approximation, then we do this through plotting the estimate

1 $$\begin{aligned} \phi (\pi ) = E \{ E(Y|A=\max (A_{min},(1-\pi )*A),W)\} \end{aligned}$$

as a function of $\pi$, where $A_{min}$ is the minimum observed value of *A* across all counties in the data and $\pi$ is interpreted as the proportional reduction in the county-specific value of the variable. To avoid extrapolation, we truncate *A* at the minimum observed value for the variable among counties. In essence, we exam the resulting predicted mean outcome across all counties as though the particular variable were reduced by $\pi$% in all counties, and all other covariates remain fixed at their observed values. This plot then provides a relevant function of the importance of the variable to the outcome.

Under several strong assumptions, including that the other covariates (the *W*, being either all other predictors or all but the ones in same sub-category) contain all the confounding information, sufficient experimentation (no positivity violations^36,37^), and independence of outcomes across counties, one could interpret (1) as identifying the marginal mean had, contrary to fact, all counties been set at the stochastic value, $\max (A_{min},(1-\pi )*A)$. We do not have information to impose a time-ordering among the covariates, so we treat these plots as a non-parametric form of association measure.

To derive inference, we use the non-parametric bootstrap (randomly sampling counties with replacement) for each $\pi$ reduced mean. The procedure is as follows: (1) fit each outcome using the aforementioned set of learners, (2) iteratively remove variables and determine risk-ratios, (3) set the variable with the highest risk-ratio as the target variable for marginal predictions, (4) for each percentage from 0 to 1.0 at 0.10 intervals (a) resample the county data with replacement, (b) refit the SuperLearner with this resampled data, (c) reduce or increase the target variable for the respective percentage, and (d) predict the expected number of cases or mortalities with this new fit. Here, in step (4) we bootstrap this procedure 1000 times by resampling, refitting, and making marginal predictions, in order to create confidence intervals (CI) at each percent change to the target variable.

Note that we use three different models to estimate the regression *E*(*Y*|*A*, *W*) depicted in (1): fully adjusted, adjusted only variables not in the sub-category of the variable of interest, and unadjusted. To evaluate the performance of our model, we compare each marginal prediction to that of a univariate model of the target variable (found by variable importance) for each outcome. Here, a generalized additive model (GAM^38^) was retrained at each iteration of the bootstrap for each reduction in the target variable and cases/mortalities were predicted through this univariate model. Likewise, to investigate possible over-corrections of our SuperLearners, we also remove similar variables that may have strong multi-collinearity with the target variable. This was done by removing variables in the target variable sub-category and training a SuperLearner on this set of covariates through the bootstrap. Results are presented as line plots and the actual observed average or sum for each outcome are plotted as horizontal lines for comparing actual outcomes to model predictions.

All data aggregation, curation, cleaning, exploratory analysis, ML pipleine, and marginal predictions were performed in R^39^. All coding scripts are available on our GitHub page for open-access and use: (https://github.com/blind-contours/Getz_Hubbard_Covid_Ensemble_ML_Public.git).

**Table 2:** Cross validated superlearner risk across COVID-19 Outcomes.

COVID-19 outcome	Model risk (per capita)	Model risk (counts)	R-squared
Day of first case	NA	159.58	0.75
COVID-19 cases at day 25	4.22 e−05	10539.64	0.59
Total COVID-19 cases to-date	5.22 e−05	13053.75	0.87
All-cause death at day 100	2.80 e−08	7.00	0.57
All-cause death at to-date	1.42 e−07	35.52	0.59

## Results

### COVID-19 outcomes and county feature distributions

There are 3142 counties in the U.S. After our data cleaning and curation process, there were 2620 counties included in the analysis and 101 county-level features. As such, our analysis covers 83% of the U.S. population as represented by counties. In these counties, as of July 15, 2020 there were 243,065 cases at day 25, 2,531,134 cases to-date, 53,018 all-cause deaths at day 100, 111,991 all-cause deaths to-date, and the average number of days to the first case in a county relative to the first case in Snohomish County, Washington was 68 days. A description of the variables used and their sources are given in supplementary materials (Table S1). A breakdown of these features with the respective mean, standard deviation, and range of values are also given in the supplementary materials (Table S2).

### Exploratory

The heatmap for exploring the patterns in these data are given in Fig. 1. The marked section of the heatmap show an outcome trend for (1) first quantile of day of first case (Q1 = earlier days of first case): (2) highest total deaths to-date (Q4): (3) highest deaths at day 100 (Q3): (4) highest total COVID-19 cases to-date (Q4): and (5) highest COVID-19 cases at day 25 (Q4). The distribution of the outcome quantiles across the county dendrogram groups are provided in the supplementary materials (Table S8). The cluster of counties with most severe outcomes is marked on the left in Fig. 1. These patterns indicate there are counties that cluster together based on similar characteristics and these counties correspond with an earlier first case in the county and higher COVID-19 case and mortality rates. For a breakdown of the number of counties in each state in this cluster see the supplementary material; briefly, however, the states with the highest number of counties in this cluster with highest outcomes are 1. Virginia (36), 2. Florida (33), and 3. Texas (30). The highest column values (red and orange pixels in the heat map) in this highest county row cluster occur in branches 3–16 and branches 46–53 of the column-wise dendrogram. A full list for each cluster is given in the plot but the highest county features for this subset were: 1—obesity, 2—sexually transmitted diseases, 3—income inequality, 4—food environment index, 5—CDC limited English scores, 6—latitude, 7—poverty income ratio, 8—GDP, 9—preventable hospital stays, 10—arthritis, 11—asthma, and 12—ischemic heart disease.

### SuperLearner

As discussed, we use cross-validation to generate a coefficient that defines the weight for a respective learner in the ensemble. This procedure is done for each outcome and the same learners are used for each outcome (outside of day of first case where Poisson outcomes were defined). Tables S3–S7 in supplementary give a detailed breakdown of how each algorithm was used in the SuperLearner, the risk of the respective algorithm and the overall risk of the SuperLearner. Overall, our SuperLearners were able to achieve good fits by utilizing multiple algorithms. Results show that for each outcome the SuperLearners were largely built from multiple elastic net models, multiple xgboost models, and random forest. Table 2 shows resulting risk for each SuperLearner for each outcome. Because the learners were fit to the per capita standardized outcome data, we multiply each risk (mean squared error or MSE) by the total population in the dataset to get absolute error based on total numbers of cases or mortalities. We also calculate the r-squared for each SuperLearner to show variance explained by each model.

### Variable importance

The top variable categories for each outcome were: (1) day of first case in a county: demography; (2) COVID-19 cases at day 25: ethnicity, transit and preventable disease; (3) total COVID-19 cases to-date: ethnicity and preventable disease; (4) mortalities at day 100: transit and ethnicity; (5) total mortalities to-date: ethnicity and transit. The top individual variables across all the models were: overall population of a county, CDC vulnerability scores for minority and limited English, public transportation use, and proportion of Black- and/or African-American individuals in a county. To visualize results, we present the risk-ratio (RR) results for each COVID-19 outcome collectively in Figs. 2 and 3 as a series of dot-plots (RR threshold at 1.01). Based on these figures, it can be seen that for day of the first case in a county the total population (RR: 1.38) was the most important variable. For per capita COVID-19 cases at day 25, the top variable is the CDC minority score (RR: 1.04). For per capita COVID-19 cases to-date, the CDC’s score for limited English speaking (1.40) the CDC’s score for minority populations (1.17) and proportion Black- and/or African-American individuals in a county (RR: 1.12) were the top variables. For per capita all-cause mortalities at day 100, proportion taking public transportation (RR: 1.14) and proportion Black- and/or African-American individuals (RR: 1.07) were the top variables. For per capita all-cause mortalities to-date, proportion Black- and/or African-American individuals (RR: 1.08) and proportion taking public transportation (RR: 1.03) were the top county features.

### Marginal prediction results

For the day of first case, population size was clearly most important predictor. For examining the association of cases and deaths of COVID-19 we choose two outcomes (total deaths and cases by July 14, 2020) and three of the most consistently important variables: two related to demographic features of the population (CDC Minority Score and Proportion of Black- and/or African-American individuals) and one related to transportation (metric of public transportation use). We estimate the relationship of proportional reductions in each of the predictor variables on the marginal outcome (using the substitution estimator of (1)) based upon a machine learning fit when controlling for: (1) all other variables (including possibly strongly collinear variables within the same sub-category), (2) only variables outside the target variable sub-category, and (3) nothing (unadjusted). The latter estimator of *E*(*Y*|*A*) is based upon a smooth regression of the outcome versus the continuous covariates, specifically using general additive model with identify link (GAM^38^).

**Figure 4:**
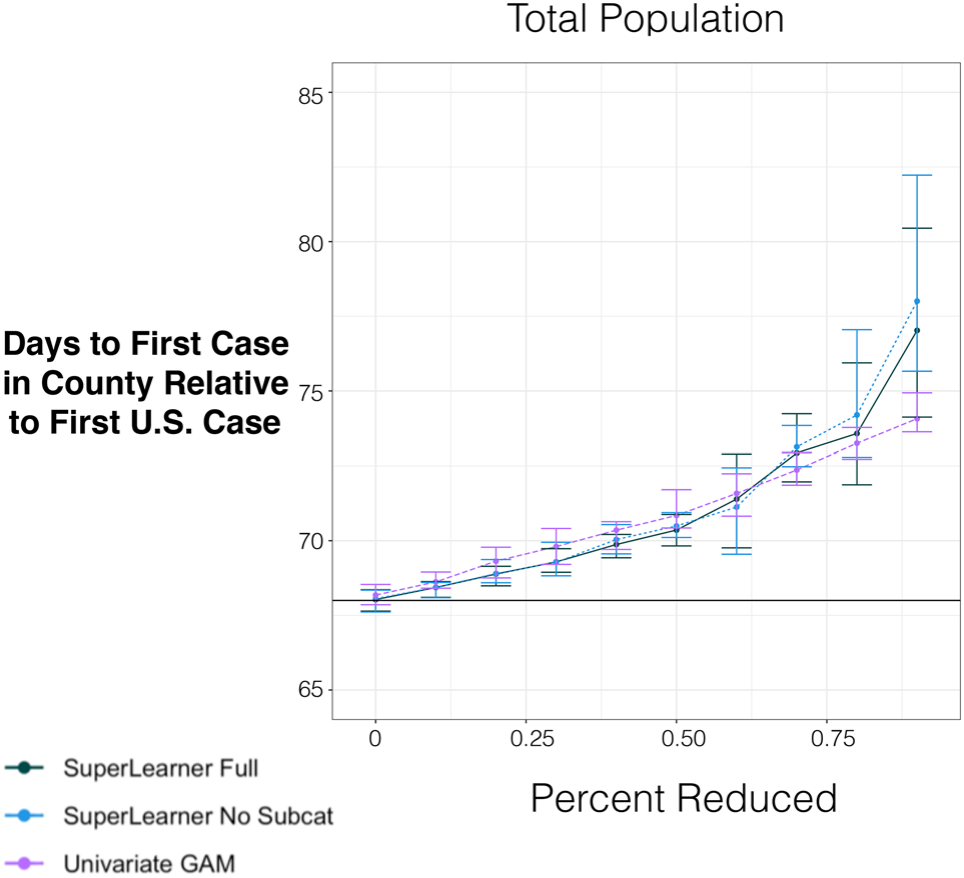
Marginal predictions of day of first case (relative to index time) for different proportional reductions of total population size for models adjusting for all other covariates, only covariates not in sub-category (see supplement Table S1) and unadjusted.

Figure 4 shows the predicted average day of first case across all counties for each proportion of population size reduced across the counties. Generally, smaller county populations are predicted to have a delay relative to counties with higher population sizes, *all other factors in the model staying constant* (the current average being 68 days after the initial U.S. case in Washington) for all three estimators, with somewhat larger effects in the adjusted models. For all models, a 0.50 proportional reduction in population size across the counties suggest a delay of around 2–3 days from the onset of the COVID-19 in the county.

**Figure 5:**
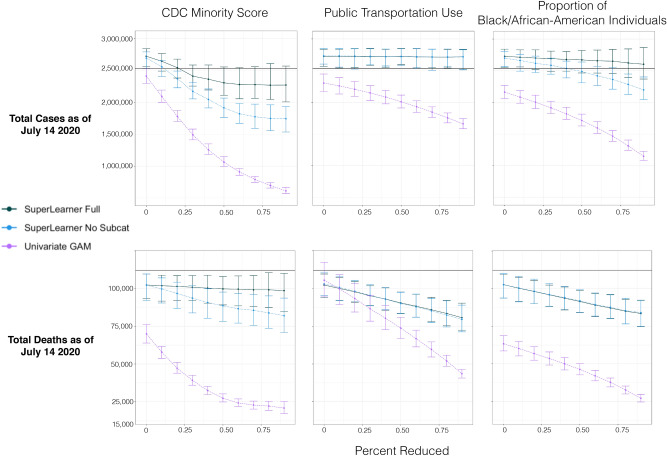
Marginal predictions of total cases and deaths by July 14, 2020) for three of the most consistently important variables in predicting the count outcomes: CDC minority score, proportion of Black- and/or African-Americans and a metric of public transportation use. X-axis is different proportional reductions of each of the three predictors, the Y-axis is the marginal predicted total counts for models adjusting for all other covariates, only covariates not in sub-category (see supplement table S1) and unadjusted. Black lines indicate actual total number of COVID-19 cases and mortalities.

Figure 5 shows plotted results for two outcomes (total counts and deaths), both versus proportion reductions in the three predictor variables. Proportional decreases in CDC Minority Score is associated with a decrease in COVID-19 cases and death. The large attenuation of the relationship in the adjusted models suggest strong confounding by the other covariates, where in the fully adjusted (and perhaps over-adjusted) curve approaches the null line. Using the curves not adjusting for other variables in the sub-category, the association suggest a 0.5 proportional reduction in the score would predict a reduction of 750,000 cases (out of around 2,400,000 total number by July 14) and approximately 10,000 deaths (out of around 115,000). Note that, particularly with deaths, the unadjusted curve is quite different from the actual number (pink line is substantially below the horizontal black line at the point of no intervention), this should be equal to that value if the model fits the data well. This is due to a few counties with extreme large counts (of both death and cases), which are poorly predicted by the bivariate smooths resulting in large positive residuals. Thus, when one estimates counts based upon reductions in the variable of interest (CDC Minority Score in this case), you get a prediction of the count that underestimates the true count. Note, by substantially reducing residual variation, the adjusted curves tend to be much closer to the observed count at 0 reduction.

Reducing public transportation suggests significant reduction in deaths, but little impact on case counts. For cases, we again have a poor fit of the bivariate smooth (unadjusted), along with the suggestion of significant confounding by other covariates. For deaths, a reduction of 50% suggest a reduction in deaths of 10,000, but the fact that the intercept for both adjusted curves (and unadjusted) is less than the observed count suggest again the influence of outliers. Bootstrapped linear regression of the marginal predictions showed that for a 10% reduction in public transit use, total deaths reduce by 2012 (95% CI [1972, 2356]).

Finally, for reductions in the proportion of Black- and/or African-American populations, there appears to be quite different estimates between the adjusted curves of COVID-19 counts, suggesting that variables in the sub-category of this variable create the possibility of over-adjustment in the full model. For deaths, the curves are nearly identical and imply that a reduction of the disparity between Black- and/or African-Americans and White-Americans in 50% of the population (one way to interpret an actual reduction in this variable) suggest a reduction of about 9000 total deaths. Likewise bootstrapped linear regression of these predictions are associated with a 10% increase in the proportion of Black and/or African-American individuals in a county increases total deaths to date by 2067 (95% CI [1189, 2654]). For total cases, although the expected COVID-19 cases was not perfectly linear given a shift in CDC minority index score, a 10% increase in CDC minority index score was associated with 111,006 additional cases (95% CI [91,991, 127,684]), using the model that adjust for variables outside of ethnicity.

## Discussion

In this paper, we took a semi-parametric (machine learning) approach to evaluating a wide range of county-level features which may impact the spread, number of cases, and deaths of COVID-19 in the U.S. Our contributions are the following: (1) curating an open-source data repository that includes variables from many sources, categorized into sub-groups and filtered such that strongly collinear variables are removed for statistical analysis; (2) demonstrating the use of these variables in ensemble machine learning to build 5 SuperLearners for each COVID-19 outcome measure; (3) evaluating the features to identify the top variables that influence each outcome; (4) adjusting the top variables in our model to make marginal predictions for each COVID-19 outcome, while controlling for all other factors to establish the strength and directionality of the relationships; (5) constructing confidence intervals around all effects via bootstrapping to evaluate significant trends from baseline and between modeling approaches. Overall, using 101 county-level features our models show very good fits to the outcomes (all observed outcomes were within model confidence intervals apart from mortality which were slightly outside our CIs at baseline).

These fits establish that our models are able to accurately predict each outcome given the county-level feature variables. Our variable importance measures for each model fit generally show a trend that the total population size drives day of first case in a county and the proportion of Black- and/or African-American individuals in a county and CDC minority scores are most important independent contributions of COVID-19 cases and deaths as of mid July.

Causal inference pertaining to the individual relationships of these variables to each outcome is speculative at best given that these study variables are ecological and also are a static snap-shot of county variables collected before the pandemic hit the U.S. However, the general trend in these results seem to represent what has been reported as the U.S. faces this continuing pandemic. That is, for day of first case in a county, the total population as the most important variable makes sense given the larger the population the higher the probability of someone being infected traveling to the respective county. Likewise, CDC minority scores and Black- and/or African-American individuals are correlated with reports that suggest that minority populations and People of Color are disproportionately impacted by COVID-19^10,12^. In addition, we also show a significant potential impact of baseline public transportation use and mortality^24,40,41^. This could indicate that there is higher probability of exposure early in the pandemic in counties where travel on public transportation leads longer and closer duration contains. This may also lead to higher infectious doses that may possibly increase severity of infection and consequent mortality, a phenomenon thought to be the case for influenza^42^.

Our finding that counties with larger CDC minority population measures have higher COVID-19 outcomes, even when controlling for 100 other county-level variables (Table 1, Table S1), show the value of such measures when trying to determine the impact of risk level based on social factors for those disproportionately impacted by COVID-19. Additional measures, however, are necessary to understand the reasons for this. For instance, although our models adjust for income, access to health, and occupation types, our data are limited to reported factors that may not account for systematic or institutional levels of cultural/societal factors placing individuals at risk. Such factors may confound or modify others for which have been adjusted and may place certain individuals at greater risk for SARS-CoV-2 infection. Our future work will integrate additional data on environmental exposures and calculate racial dissimilarity scores to further investigate findings found in this study. The main difference between our findings and those reported to date is that our analysis controls for many other possible mediating factors (e,g., access to health-care, smoking, diabetes, heart-disease, and food security).

## Conclusion

The goal of our study is to identify, early in the COVID-19 pandemic, the most salient factors that put populations at risk for COVID-19, thereby providing some guidance to individuals making difficult policy decisions at this critical time to quell the evolving pandemic. Specifically, racial composition of counties and intensity of public transportation use therein seem to be the most important risks factors for both the initial rapid growth and subsequent high incidence, and also help explain variations in mortality rates across counties. More work, however, is needed to establish causal rather than purely statistical relationships. Future work with detailed individual data will be important for getting more robust estimates of the individual impact of the factors examined. Whether causal or statistical, these results should be taken into account when developing policies for lifting restrictions. Additionally, as efforts continue to disseminate services and funding, and to roll out vaccination programs, once effective vaccines have been developed, consideration of these factors will facilitate the efficacious allocation of resources to the benefit of the US population as a whole.

## Supplementary Information

Supplementary Information 1.

## Data Availability

All code for collecting data, collected data, statistical scripts, up-to-date outcome data, visualizations, and statistical results are available on GitHub: https://github.com/blind-contours/Getz_Hubbard_Covid_Ensemble_ML_Public.git
