# Supplementary material for: Ensemble machine learning of factors influencing COVID-19 across US counties
Source: Sci Rep. 2021 Jun 3;11:11777. doi: 10.1038/s41598-021-90827-x (PMC8175420; doi:10.1038/s41598-021-90827-x)
Supplement: Supplementary file 1 — Supplementary Information 1. [file 41598_2021_90827_MOESM1_ESM.pdf]

Supplementary materials for Ensemble Machine Learning of Factors Influencing COVID-19 Across US Counties: Supplemental Document

DAVID MCCOY, WHITNEY MGBARA, NIR HORVITZ, WAYNE M. GETZ, ALAN HUBBARD

Because of the diverse number of variables and sources of information we provide this supplementary document that explains the variable aggregation methods and gives a breakdown of the variable types and distributions. Additionally, we give metrics on the fit of each of our SuperLearners calculated through cross-validation as well as details on the heatmap presented in the main body of the manuscript.

1. DATA DICTIONARY

This table gives a detail for each variable used in modeling, its source, and a descriptions:

Variable Name / Measure	Gen. Data	Category	Sub-category	Definition	Source
FIPS	no	identifier		FIPS code to identify geographic area (county); integer	
County Relative Day 25 Cases	yes	outcome		Number of cumulative cases on day 25 of the outbreak by county per capita	USAFacts
Total Cases Up To Date	yes	outcome		Number of total cumulative cases to date per capita	USAFacts
US Relative Day 100 Deaths	yes	outcome		Number of cumulative deaths on day 100 of the outbreak per county per capita	USAFacts
Total Deaths Up To Date	yes	outcome		Number of total cumulative deaths up to date per capita	USAFacts
First Case Day	yes	outcome		Day of the first case starting after Jan. 21 2020	USAFacts
Population	no	demography	demography	Adjusted population size, 2020	USAFacts
GDP	no	demography	demography	Real gross domestic product by County, 2018 from December 12, 2019 release	Bureau of Economic Analysis (BEA)

Percent Female 2018	yes	demography	demography	Percentage of the population that identifies as female, 2014-2018	5-Year American Community Survey (ACS), Sex by, Age, 2014 - 2018
Percent Black 2018	yes	demography	ethnicity	Percentage of the population that identifies as Black or African American alone, 2014-2018	5-Year American Community Survey (ACS), Race, 2014 -2018
Percent American Indian Alaskan Native 2018	yes	demography	ethnicity	Percentage of the population that identifies as American Indian and Alaska Native alone, 2014-2018	5-Year American Community Survey (ACS), Race, 2014 - 2018
Percent Asian 2018	yes	demography	ethnicity	Percentage of the population that identifies as Asian alone, 2014-2018	5-Year American Community Survey (ACS), Race, 2014 - 2018
Percent Hawaiian or Pacific Islander 2018	yes	demography	ethnicity	Percentage of the population that identifies as Native Hawaiian and Other Pacific Islander alone, 2014-2018	5-Year American Community Survey (ACS), Race, 2014 - 2018
Percent Other Race 2018	yes	demography	ethnicity	Percentage of the population that identifies as Some other race alone, 2014-2018	5-Year American Community Survey (ACS), Race, 2014 - 2018
Percent 2 or More Races 2018	yes	demography	ethnicity	Percentage of the population that identifies as two or more races alone, 2014-2018	5-Year American Community Survey (ACS), Race, 2014 - 2018
Percent Hispanic or Latino 2018	yes	demography	ethnicity	Percentage of the population that identifies as hispanic or Latino, 2014-2018	5-Year American Community Survey (ACS), Hispanic or Latino Origin by Race, 2014 - 2018
Latitude	yes	geography	location	Latitude for mean position of all the points in the geometric object determined by legal boundaries for a county based on the 2019 Census Bureau's MAF/TIGER database for use with Esri's ArcGIS	TIGER/Line Geodatabases
Longtitude	yes	geography	location	Longitude for mean position of all the points in the geometric object determined by legal boundaries for a county based on the 2019 from the Census Bureau's MAF/TIGER database for use with Esri's ArcGIS	TIGER/Line Geodatabases
Nearest Airport Distance	yes	geography	airport	Distance from county centroid to the nearest airport (km)	TIGER/Line Geodatabases;

Federal Aviation Administration (FAA)					
Nearest Airport Enplanement	yes	geography	airport	Number of enplanments for the nearest airport to the county centroid, 2018	TIGER/Line Geodatabases;
Federal Aviation Administration (FAA)					
Nearest Airport 50	yes	geography	airport	Distance from county centroid to the nearest airport that has 50,000,000 or more enplanments (km)	TIGER/Line Geodatabases;
Federal Aviation Administration (FAA)					
Nearest Airport Enplanement 50	yes	geography	airport	Number of enplanments for the nearest airport to the county centroid that has 50,000,000 or more enplanements, 2018	TIGER/Line Geodatabases Federal Aviation Administration (FAA)
Land Area	yes	geography	geography	Area of the county geometry from 2019 tiger census shapefile (square km)	TIGER/Line Geodatabases
Urban Rural Status	no	healthcare	healthcare	Number of Hospitals by county, 2017	Interactive Atlas of Heart Disease and Stroke (2014- 2016)
All Stroke Death Rate	no	health	disease prev	Stroke Death Rate per 100,000, 35+, All Races/Ethnicities, Both Genders, 2016-2018	Interactive Atlas of Heart Disease and Stroke (2014- 2016)
Pemature Death Rate	no	health	disease prev	Years of potential life lost before age 75 per 100,000 population (age-adjusted)	County Health Rankings Roadmaps
Adule Smoking	no	health	disease prev	Percentage of adults who are current smokers.	County Health Rankings Roadmaps
Adult Obesity	no	health	disease prev	Percentage of the adult population (age 20 and older) that reports a body mass index (BMI) greater than or equal to 30 kg/m^2	County Health Rankings Roadmaps
Healthy Food Env.	no	health	health envir.	Index of factors that contribute to a healthy food environment, from 0 (worst) to 10 (best).	County Health Rankings Roadmaps
Access to Exercise Opportunities	no	health	health envir.	Percentage of population with adequate access to locations for physical activity.	County Health Rankings Roadmaps
Excessive Drinking	no	health	health envir.	Percentage of adults reporting binge or heavy drinking.	County Health Rankings Roadmaps
HIV Prevalence		health	disease prev		
Sexually Transmitted Diseases	no	health	disease prev	Number of newly diagnosed chlamydia cases per 100,000 population	County Health Rankings Roadmaps

Life Expectancy	no	health	health envir.	Average number of years a person can expect to live.	County Health Rankings Roadmaps
Food Insecurity Index	no	health	health envir.	Percentage of population who lack adequate access to food.	County Health Rankings Roadmaps
Prevalence of Alzheimer's and Dimentia over 65	no	health	disease prev	Prevalence of Alzheimer's Disease and Related Dementia, All Fee-for-Service Beneficiaries, 2017 (%)	Centers for Medicare & Medicaid Services (CMS)
Prevalence Arthritis	no	health	disease prev	Prevalence of Arthritis (Osteoarthritis and Rheumatoid), All Fee-for-Service Beneficiaries, 2017 (%)	Centers for Medicare & Medicaid Services (CMS)
Prevalence Asthma	no	health	disease prev	Prevalence of Asthma, All Fee-for-Service Beneficiaries, 2017 (%)	Centers for Medicare Medicaid Services (CMS)
Prevalence Cancer	no	health	disease prev	Prevalence of Cancer (Breast, Colorectal, Lung, and Prostate), All Fee-for-Service Beneficiaries, 2017 (%)	Centers for Medicare & Medicaid Services (CMS)
Prevalence Chronic Kidney Disease	no	health	disease prev	Prevalence of Chronic Kidney Disease, All Fee-for- Service Beneficiaries, 2017 (%)	Centers for Medicare Medicaid Services (CMS)
Prevalence COPD	no	health	disease prev	Prevalence of Chronic Obstructive Pulmonary Disease, All Fee-for-Service Beneficiaries, 2017 (%)	Centers for Medicare Medicaid Services (CMS)
Prevalence Depression	no	health	disease prev	Prevalence of Depression, All Fee-for-Service Beneficiaries, 2017 (%)	Centers for Medicare Medicaid Services (CMS)
Prevalence Diabetes	no	health	disease prev	Prevalence of Diabetes, All Fee-for-Service Beneficiaries, 2017 (%)	Centers for Medicare Medicaid Services (CMS)
Prevalence Drug Abus	no	health	disease prev	Prevalence of Drug Abuse/ Substance Abuse, All Fee-for-Service Beneficiaries, 2017 (%)	Centers for Medicare Medicaid Services (CMS)
Prevalence Hyperlipidemia	no	health	disease prev	Prevalence of Hyperlipidemia (High cholesterol), All Fee-for-Service Beneficiaries, 2017 (%)	Centers for Medicare Medicaid Services (CMS)
Prevalence Hypertension	no	health	disease prev	Prevalence of Hypertension (High blood pressure), All Fee-for-Service Beneficiaries, 2017 (%)	Centers for Medicare Medicaid Services (CMS)
Prevalence Ischemic Heart Disease	no	health	disease prev	Prevalence of Ischemic Heart Disease, All Fee-for- Service Beneficiaries, 2017 (%)	Centers for Medicare Medicaid Services (CMS)
Prevalence Osteoporosis	no	health	disease prev	Prevalence of Osteoporosis, All Fee-for-Service Beneficiaries, 2017 (%)	Centers for Medicare &

Medicaid Services (CMS)					
Prevalence Schizophrenia	no	health	disease prev	Prevalence of Schizophrenia and Other Psychotic Disorders, All Fee-for-Service Beneficiaries, 2017 (%)	Centers for Medicare & Medicaid Services (CMS)
Number of Hospital	no	healthcare	healthcare	Number of Hospitals by county, 2017	Interactive Atlas of Heart Disease and Stroke (2014-
2016)					
Percentage Uninsured	no	healthcare	healthcare	Percentage of the population under age 65 that has no health insurance coverage	County Health Rankings Roadmaps
Primary Care Physicians	no	healthcare	healthcare	Number of primary care physicians per 100,000 population	County Health Rankings Roadmaps
Preventable Hospital Stays	no	healthcare	healthcare	Hospital discharge rate for ambulatory care-sensitive conditions per 100,000 fee-forservice Medicare enrollees	County Health Rankings Roadmaps
Flu Vaccinations	no	healthcare	healthcare	Percentage of fee-for-service Medicare enrollees that had an annual flu vaccination	County Health Rankings Roadmaps
Percentage Household Overcrowding	no	housing	density	Percentage of households with overcrowding – more than 1 person per room	Health Rankings Roadmaps
Percent Labor Force	yes	occupation	occupation	Percentage of population employed in labor force, 2014-2018	5-Year American Community Survey (ACS), Health Insurance Coverage Status and Type by Employment Status, 2014 - 2018
Park Access	no	physical environment	physical environment	Percentage of Population Living Within Half a Mile of a Park, 2015	Interactive Atlas of Heart Disease and Stroke (2014-
2016)					
Air Pollution	no	physical environment	physical environment	Average daily density of fine particulate matter in micrograms per cubic meter (PM2.5) in a county	County Health Rankings & Roadmaps
Drinking Water Quality	no	physical environment	physical environment	Indicator of the presence of health-related drinking water violations. 0=No, 1=Yes	County Health Rankings & Roadmaps
Precipitation	no	physical environment	physical environment	Precipitation in January 2020 (in)	National Centers for Environmental Information / National Oceanic and Atmospheric Administration
(NOAA)					

High School Graduation	no	social	education	Percentage of ninth-grade cohort that graduates in four years.	County Health Rankings Roadmaps
Some College Level	no	social	education	Percentage of adults ages 25-44 with some post- secondary education.	County Health Rankings Roadmaps
Unemployment	no	social	occupation	Percentage of the civilian labor force, age 16 and older, that is unemployed but seeking work	County Health Rankings Roadmaps
Income Inequality	no	social	economic	Ratio of household income at the 80th percentile to income at the 20th percentile.	County Health Rankings Roadmaps
Median Household Income	no	social	economic	The income where half of households in a county earn more and half of households earn less.	County Health Rankings Roadmaps
Per Capita Income	no	social	economic	Percentile per capita income estimate	CDC's Social Vulnerability
Index (SVI)					
Percent People over 65	no	demography	age	Percentile percentage of persons aged 65 and older estimat	CDC's Social Vulnerability
Index (SVI)					
Percent People below 17	no	demography	age	Percentile percentage of persons aged 17 and younger estimate	CDC's Social Vulnerability
Index (SVI)					
Percent Disability Individuals	no	health	disease prev	Percentile percentage of civilian noninstitutionalized population with a disability estimate	CDC's Social Vulnerability
Index (SVI)					
Percent Single Parent	no	social	economic	Percentile percentage of single parent households with children under 18 estimate	CDC's Social Vulnerability
Index (SVI)					
Percent Minority	no	social	Ethnicity	Percentile percentage minority (all persons except white, non - Hispanic) estimate	CDC's Social Vulnerability
Index (SVI)					
Percent Limited English	no	social	Ethnicity	Percentile percentage of persons (age 5+) who speak English "less than well" estimate	CDC's Social Vulnerability
Index (SVI)					
Percent Multi-Unit Buildings	no	social	economic	Percentile percentage housing in structures with 10 or more units estimate	CDC's Social Vulnerability
Index (SVI)					

Percent Mobile Homes	no	social	economic	Percentile percentage mobile homes estimate	CDC's Social Vulnerability
Index (SVI)					
Percent No Vehicle	no	social	economic	Percentile percentage households with no vehicle available estimate	CDC's Social Vulnerability
Index (SVI)					
Percent Institutionalized Individuals	no	social	density	Percentile percentage of persons in institutionalized group quarters estimate	CDC's Social Vulnerability
Index (SVI)					
Percent No Workers in Household	yes	social	economic	Percentage of the households with no workers, 2014 2018	5-Year American Community Survey (ACS), Household size by number of workers in household, 2014 - 2018
Percent with Poverty Ratio 100	yes	social	economic	Percentage of population with a ratio of income to poverty level in the past 12 months under 1.00, 2014 2018	5-Year American Community Survey (ACS), Ratio of income to poverty levels in the Past 12 Months, 2014 -
2018 Percent with Poverty Ratio 100-200	yes	social	economic	Percentage of population with a ratio of income to poverty level in the past 12 months from 1.00 to 2.00, 2014-2018	5-Year American Community Survey (ACS), Ratio of income to poverty levels in the Past 12 Months, 2014 -
2018					
Driving Alone to Work					
Percent Taking Public Transportation	yes	transit	transit	Percentage of the population with public transportation (excluding taxicab) as means of transportation to work, 2014-2018	5-Year American Community Survey (ACS), Means of Transportation to Work,
2014 - 2018					
Resident Workplace Commute Flow	no	transit	transit	Residence County to Workplace County Commuting Flows by Residence	5-Year American Community Survey (ACS), Commuting Flows, 2011-2015
All Federal Occupations		occupation	occupation	10 Total, all industries federal	Quarterly Census of Employment and Wages
All State Occupations		occupation	occupation	10 Total, all industries state	Quarterly Census of Employment and Wages
All Local Occupations		occupation	occupation	10 Total, all industries local	Quarterly Census of Employment and Wages

Goods producing Labor	occupation	occupation	101 Goods-producing	Quarterly Census of Employment and Wages
Natural Mining and Farming Labor	occupation	occupation	1011 Natural resources and mining	Quarterly Census of Employment and Wages
Construction Labor	occupation	occupation	1012 Construction	Quarterly Census of Employment and Wages
Manufacturing Labor	occupation	occupation	1013 Manufacturing	Quarterly Census of Employment and Wages
Service Industry Labor	occupation	occupation	102 Service-providing	Quarterly Census of Employment and Wages
Trade and Transit Labor	occupation	occupation	1021 Trade, transportation, and utilities	Quarterly Census of Employment and Wages
Information Service Labor	occupation	occupation	1022 Information	Quarterly Census of Employment and Wages
Financial Labor	occupation	occupation	1023 Financial activities	Quarterly Census of Employment and Wages
Professional Business Laor	occupation	occupation	1024 Professional and business services	Quarterly Census of Employment and Wages
Education and Health Labor	occupation	occupation	1025 Education and health services	Quarterly Census of Employment and Wages
Leisure Labor	occupation	occupation	1026 Leisure services	Quarterly Census of Employment and Wages
Ratio of Republicans	social	demography	ratio of republican votes to total votes in 2016 election	MIT election lab
Social Associations	social	density	social associates per 10k	County Health Rankings & Roadmaps
Percent with Mental Disorder	social	disease prev	Proportion reporting mental disorders	County Health Rankings & Roadmaps
Percent Insufficient Sleep	social	disease prev	Proportion reporting sleep deprivation	County Health Rankings & Roadmaps
Commute Grocery or Pharmacy	transit	transit	google mobility data to grocery stores and pharmacies	Google
Commute Parks	transit	transit	google mobility data to parks	Google
Commute Residential	transit	transit	google mobility data to residential areas	Google
Commute Retail and Rec.	transit	transit	google mobility data to retail and recreations	Google

Commute Transit Stations	transit	transit	google mobility data to transit stations	Google
Commute Workplaces	transit	transit	google mobility data to workplaces	Google

Table S1. Variables used in modeling with sources, sub-categories and details

2. STATISTICAL BREAKDOWN OF VARIABLES USED

Variable Name / Measure	Variable Type	Minimum	Maximum	Mean	Standard Deviation	Median
FIPS	Identifier	10001.0000	56045.0000	33139.3309	13169.5363	31170.0000
County Relative Day 25 Cases	Outcome	1.0000	13869.0000	92.7729	518.7487	13.0000
Total Cases Up To Date	Outcome	1.0000	95557.0000	966.0817	4184.6724	109.0000
US Relative Day 100 Deaths	Outcome	0.0000	5228.0000	20.2359	185.0201	0.0000
Total Deaths Up To Date	Outcome	0.0000	7171.0000	42.7447	288.2969	2.0000
First Case Day	Outcome	1.0000	148.0000	67.9084	17.3803	64.0000
Population	Predictor	625.0000	5150233.0000	95405.6099	258464.9638	27036.5000
GDP	Predictor	26968.0000	711974400.0000	5743017.6373	23894006.9611	1084813.5000
Percent Female 2018	Predictor	0.2100	0.5792	0.4994	0.0226	0.5038
Percent Black 2018	Predictor	0.0000	0.8741	0.0877	0.1393	0.0237
Percent American Indian Alaskan Native 2018	Predictor	0.0000	0.8553	0.0168	0.0665	0.0033
Percent Asian 2018	Predictor	0.0000	0.2526	0.0118	0.0194	0.0059
Percent Hawaiian or Pacific Islander 2018	Predictor	0.0000	0.0214	0.0006	0.0015	0.0002
Percent Other Race 2018	Predictor	0.0000	0.5701	0.0190	0.0355	0.0083
Percent 2 or More Races 2018	Predictor	0.0000	0.1898	0.0227	0.0168	0.0192
Percent Hispanic or Latino 2018	Predictor	0.0000	0.9907	0.0869	0.1334	0.0383
Latitude	Predictor	25.0468	48.8296	38.4292	4.8951	38.6032
Longtitude	Predictor	-124.2152	-67.6091	-90.4847	10.7467	-89.1648
Nearest Airport Distance	Predictor	0.8232	259.7739	63.7218	34.4058	59.6057
Nearest Airport Enplanement	Predictor	2636.0000	51865797.0000	2459136.1817	7337672.2813	178057.0000
Nearest Airport 50	Predictor	2.3659	940.4769	237.4400	140.2948	216.9125
Nearest Airport Enplanement 50	Predictor	5790847.0000	51865797.0000	17699825.2695	12418360.1454	15292670.0000

Land Area	Predictor	58690498.0000	47090939040.0000	2243334237.1412	2886455081.9736	1493745477.5000
Urban Rural Status	Predictor	1.0000	4.0000	3.4702	0.7729	4.0000
All Stroke Death Rate	Predictor	27.9000	180.2000	76.9035	15.4610	75.8000
Pemature Death Rate	Predictor	0.0014	14.8514	0.5859	0.9028	0.3116
Adule Smoking	Predictor	0.0591	0.4149	0.1758	0.0349	0.1703
Adult Obesity	Predictor	0.1240	0.5770	0.3314	0.0519	0.3330
Healthy Food Env.	Predictor	0.0000	10.0000	7.5320	1.0571	7.7000
Access to Exercise Opportunities	Predictor	0.0000	1.0000	0.6264	0.2231	0.6574
Excessive Drinking	Predictor	0.0781	0.2862	0.1759	0.0312	0.1760
HIV Prevalence	Predictor	0.0000	0.2484	0.0074	0.0141	0.0048
Sexually Transmitted Diseases	Predictor	0.0001	3.2434	0.0219	0.0740	0.0110
Life Expectancy	Predictor	61.6256	89.4894	77.4182	2.7707	77.4617
Food Insecurity Index	Predictor	0.0290	0.3630	0.1313	0.0383	0.1270
Prevalence of Alzheimer's and Dimentia over 65	Predictor	4.1825	30.4888	11.8557	1.9348	11.8481
Prevalence Arthritis	Predictor	18.7000	62.7000	33.2102	4.9535	33.3000
Prevalence Asthma	Predictor	1.4000	11.6000	4.4356	1.1254	4.4000
Prevalence Cancer	Predictor	3.5000	12.1000	7.5025	1.2220	7.5000
Prevalence Chronic Kidney Disease	Predictor	9.2000	51.5000	23.1285	4.3022	23.1000
Prevalence COPD	Predictor	3.6000	32.1000	13.0471	3.6783	12.6000
Prevalence Depression	Predictor	7.2000	35.9000	17.8434	3.3333	17.8000
Prevalence Diabetes	Predictor	8.5000	49.6000	27.2415	4.7142	27.3000
Prevalence Drug Abus	Predictor	0.0000	16.7000	3.3254	1.6711	3.1000
Prevalence Hyperlipidemia	Predictor	10.3000	67.6000	38.6523	8.5261	39.9000
Prevalence Hypertension	Predictor	28.8000	74.9000	57.0606	8.1435	58.7000
Prevalence Ischemic Heart Disease	Predictor	13.9000	46.9000	27.1162	5.1658	26.8000
Prevalence Osteoporosis	Predictor	1.1000	16.6000	5.4366	1.5669	5.3000
Prevalence Schizophrenia	Predictor	0.0000	17.5000	2.7311	1.0922	2.6000
Number of Hospital	Predictor	0.0000	48.0000	1.3912	2.0305	1.0000
Percentage Uninsured	Predictor	0.0226	0.3375	0.1148	0.0527	0.1050
Primary Care Physicians	Predictor	0.0000	0.0051	0.0005	0.0003	0.0005

Preventable Hospital Stays	Predictor	0.0010	5.8528	0.3379	0.4999	0.1687
Flu Vaccinations	Predictor	0.0700	0.6600	0.4237	0.0947	0.4400
Percentage Household Overcrowding	Predictor	0.0000	0.1694	0.0225	0.0172	0.0184
Percent Labor Force	Predictor	0.3759	0.9210	0.7118	0.0794	0.7181
Park Access	Predictor	0.0000	100.0000	19.0836	18.1604	14.0000
Air Pollution	Predictor	3.0000	15.0000	9.1167	1.8303	9.4000
Drinking Water Quality	Predictor	0.0000	1.0000	0.3656	0.4817	0.0000
Precipitation	Predictor	0.0100	29.4900	3.5264	2.9479	3.1550
High School Graduation	Predictor	0.4040	1.0000	0.8901	0.0669	0.8995
Some College Level	Predictor	0.1518	0.9034	0.5770	0.1165	0.5779
Unemployment	Predictor	0.0130	0.1326	0.0409	0.0134	0.0386
Income Inequality	Predictor	2.5431	11.9706	4.4894	0.7311	4.3797
Median Household Income	Predictor	25973.0000	140382.0000	52705.2481	13257.0179	50823.5000
Per Capita Income	Predictor	-999.0000	0.9997	0.1197	19.5289	0.4954
Percent People over 65	Predictor	0.0000	1.0000	0.4961	0.2816	0.4893
Percent People below 17	Predictor	0.0003	0.9997	0.4998	0.2860	0.5040
Percent Disability Individuals	Predictor	0.0006	1.0000	0.4954	0.2820	0.4906
Percent Single Parent	Predictor	0.0035	0.9997	0.4990	0.2794	0.4900
Percent Minority	Predictor	0.0010	1.0000	0.4789	0.2865	0.4691
Percent Limited English	Predictor	0.0000	1.0000	0.4725	0.2954	0.4642
Percent Multi-Unit Buildings	Predictor	0.0000	1.0000	0.4944	0.2853	0.4947
Percent Mobile Homes	Predictor	0.0000	1.0000	0.5005	0.2869	0.4963
Percent No Vehicle	Predictor	0.0000	0.9997	0.4948	0.2836	0.5002
Percent Institutionalized Individuals	Predictor	0.0000	1.0000	0.4935	0.2924	0.4995
Percent No Workers in Household	Predictor	0.1084	0.7108	0.3176	0.0762	0.3105
Percent with Poverty Ratio 100	Predictor	0.0230	0.5510	0.1556	0.0639	0.1462
Percent with Poverty Ratio 100-200	Predictor	0.0587	0.5277	0.2494	0.0614	0.2479
Driving Alone to Work	Predictor	0.0605	0.9517	0.8052	0.0596	0.8136

Percent Taking Public Transportation	Predictor	0.0000	0.6136	0.0085	0.0302	0.0032
Resident Workplace Commute Flow	Predictor	0.1625	0.6219	0.4251	0.0623	0.4273
All Federal Occupations	Predictor	0.0000	0.0112	0.0006	0.0006	0.0004
All State Occupations	Predictor	0.0000	0.0048	0.0006	0.0005	0.0005
All Local Occupations	Predictor	0.0000	0.0176	0.0015	0.0014	0.0011
Goods producing Labor	Predictor	0.0002	0.0653	0.0054	0.0033	0.0044
Natural Mining and Farming Labor	Predictor	0.0000	0.0610	0.0016	0.0025	0.0008
Construction Labor	Predictor	0.0002	0.0295	0.0026	0.0016	0.0023
Manufacturing Labor	Predictor	0.0001	0.0075	0.0011	0.0006	0.0010
Service Industry Labor	Predictor	0.0027	0.0858	0.0194	0.0073	0.0182
Trade and Transit Labor	Predictor	0.0007	0.0190	0.0058	0.0022	0.0054
Information Service Labor	Predictor	0.0000	0.0032	0.0004	0.0003	0.0003
Financial Labor	Predictor	0.0001	0.0136	0.0021	0.0010	0.0020
Professional Business Laor	Predictor	0.0001	0.0272	0.0032	0.0019	0.0027
Education and Health Labor	Predictor	0.0002	0.0326	0.0032	0.0024	0.0027
Leisure Labor	Predictor	0.0001	0.0161	0.0025	0.0015	0.0023
Ratio of Republicans	Predictor	0.0946	0.9458	0.6388	0.1467	0.6653
Social Associations	Predictor	0.0000	52.3139	11.9612	5.5981	11.4235
Percent with Mental Disorder	Predictor	8.0032	21.0132	12.9910	1.9344	12.9401
Percent Insufficient Sleep	Predictor	23.2268	46.7078	33.1343	4.0185	33.0519
Commute Grocery or Pharmacy	Predictor	-0.6800	0.0011	-0.2002	0.1421	-0.2107
Commute Parks	Predictor	-1.5902	0.8783	-0.0650	0.5552	0.0766
Commute Residential	Predictor	0.3165	0.6401	0.4380	0.0721	0.4271
Commute Retail and Rec.	Predictor	-1.7072	-0.9861	-1.2988	0.1833	-1.2733
Commute Transit Stations	Predictor	-1.9894	-0.4005	-1.1413	0.3867	-1.1984
Commute Workplaces	Predictor	-1.4059	-0.7389	-1.0598	0.1442	-1.0743

Table S2. Range, mean, median, standard deviation and variable type for variables used

3. CROSS VALIDATED RISK OF THE ESTIMATORS

Learner	Coefficient	Mean Risk	SE Risk	Fold SD	Fold Min Risk	Fold Max Risk
Conditional Mean	0.00	326.50	16.83	21.96	303.41	354.08
Poisson Xgboost depth = 5, rounds = 100	0.08	195.00	10.83	26.21	175.12	239.99
Poisson Xgboost depth = 10, rounds = 200	0.12	201.06	11.25	22.38	181.37	234.70
Poisson Ridge Regression	0.07	203.47	11.86	18.99	181.00	230.33
Poisson Lasso Regression	0.00	227.91	29.42	59.21	185.14	331.88
Poisson Gradient Boosting Machine	0.72	177.07	9.83	15.58	165.87	203.52
Elastic Net, alpha = 0.25	0.01	210.91	18.95	38.65	179.15	274.90
Elastic Net, alpha = 0.50	0.00	220.47	24.80	51.71	179.24	306.17
Elastic Net, alpha = 0.75	0.00	224.28	28.53	60.82	178.31	327.66
SuperLearner	NA	178.00	9.98	16.26	162.74	204.02

Table S3. Cross-Validated Coefficients and Risk for Day of First Case Outcome

Learner	Coefficient	Mean Risk	SE Risk	Fold SD	Fold Min Risk	Fold Max Risk
GLM	0.00	124.28	123.25	275.33	0.37	616.80
Conditional Mean	0.25	1.10	0.37	1.23	0.35	3.26
Ridge Regression	0.00	48.01	46.99	104.79	0.32	235.46
Elastic Net	0.00	36.60	35.58	79.28	0.32	178.42
Lasso Regression	0.00	30.88	29.86	66.49	0.31	149.79
Xgboost, nrounds = 50, depth =2, eta =0.001	0.00	54800.39	9.26	31.51	54748.31	54831.68
Xgboost, nrounds = 50, depth =4, eta =0.001	0.00	54800.39	9.26	31.51	54748.31	54831.68
Xgboost, nrounds = 50, depth =6, eta =0.001	0.00	54800.39	9.26	31.51	54748.31	54831.68
Xgboost, nrounds = 50, depth =8, eta =0.001	0.00	54800.39	9.26	31.51	54748.31	54831.68
Xgboost, nrounds = 50, depth =8, eta =0.01	0.00	22179.65	5.79	21.39	22144.36	22201.03
Random Forest, ntrees = 10	0.25	1.07	0.34	1.10	0.44	3.03
Xgboost, nrounds = 50, depth =4, eta =0.2	0.25	1.20	0.35	1.01	0.47	2.99
Xgboost, nrounds = 50, depth =4, eta =0.3	0.25	1.34	0.36	0.98	0.62	3.05
SuperLearner	NA	13.23	12.20	26.95	0.49	61.40

Table S4. Cross-Validated Coefficients and Risk for Number of Cases at Day 25

Learner	Coefficient	Mean Risk	SE Risk	Fold SD	Fold Min Risk	Fold Max Risk
GLM	0.00	416.94	293.75	698.71	66.12	1664.87
Conditional Mean	0.00	182.98	23.67	56.45	108.74	247.13
Ridge Regression	0.13	120.26	21.51	54.20	62.28	194.47
Elastic Net	0.01	142.90	31.48	100.09	61.35	310.72
Lasso Regression	0.01	141.39	31.47	100.07	60.23	310.72
Xgboost, nrounds = 50, depth =2, eta =0.001	0.00	586264.00	387.48	1041.23	585286.52	587872.96
Xgboost, nrounds = 50, depth =4, eta =0.001	0.00	586264.00	387.48	1041.23	585286.52	587872.96
Xgboost, nrounds = 50, depth =6, eta =0.001	0.00	586264.00	387.48	1041.23	585286.52	587872.96
Xgboost, nrounds = 50, depth =8, eta =0.001	0.00	586264.00	387.48	1041.23	585286.52	587872.96
Xgboost, nrounds = 50, depth =8, eta =0.01	0.00	237383.68	240.87	704.53	236711.62	238471.76
Random Forest, ntrees = 10	0.23	110.35	20.25	43.24	71.89	180.76
Xgboost, nrounds = 50, depth =4, eta =0.2	0.32	101.36	18.00	36.39	69.40	157.70
Xgboost, nrounds = 50, depth =4, eta =0.3	0.30	113.92	18.48	35.33	78.80	168.75
SuperLearner	NA	103.72	18.92	46.89	61.26	175.62

Table S5. Cross-Validated Coefficients and Risk for Total Cases to-date

Learner	Coefficient	Mean Risk	SE Risk	Fold SD	Fold Min Risk	Fold Max Risk
GLM	0.13	0.06	0.06	0.13	0.00	0.30
Conditional Mean	0.13	0.00	0.00	0.00	0.00	0.00
Ridge Regression	0.13	0.00	0.00	0.00	0.00	0.00
Elastic Net	0.13	0.00	0.00	0.00	0.00	0.00
Lasso Regression	0.13	0.00	0.00	0.00	0.00	0.00
Xgboost, nrounds = 50, depth =2, eta =0.001	0.00	11989.94	0.18	0.58	11989.25	11990.56
Xgboost, nrounds = 50, depth =4, eta =0.001	0.00	11989.94	0.18	0.58	11989.25	11990.56
Xgboost, nrounds = 50, depth =6, eta =0.001	0.00	11989.94	0.18	0.58	11989.25	11990.56
Xgboost, nrounds = 50, depth =8, eta =0.001	0.00	11989.94	0.18	0.58	11989.25	11990.56
Xgboost, nrounds = 50, depth =8, eta =0.01	0.00	4852.61	0.12	0.40	4852.14	4853.04
Random Forest, ntrees = 10	0.12	0.00	0.00	0.00	0.00	0.00
Xgboost, nrounds = 50, depth =4, eta =0.2	0.12	0.00	0.00	0.00	0.00	0.00
Xgboost, nrounds = 50, depth =4, eta =0.3	0.13	0.00	0.00	0.00	0.00	0.00
SuperLearner	NA	0.00	0.00	0.00	0.00	0.01

Table S6. Cross-Validated Coefficients and Risk for Deaths at day 100

Learner	Coefficient	Mean Risk	SE Risk	Fold SD	Fold Min Risk	Fold Max Risk
GLM	0.13	0.06	0.06	0.13	0.00	0.30
Conditional Mean	0.13	0.00	0.00	0.00	0.00	0.00
Ridge Regression	0.13	0.00	0.00	0.00	0.00	0.00
Elastic Net	0.13	0.00	0.00	0.00	0.00	0.00
Lasso Regression	0.13	0.00	0.00	0.00	0.00	0.00
Xgboost, nrounds = 50, depth =2, eta =0.001	0.00	11989.94	0.18	0.58	11989.25	11990.56
Xgboost, nrounds = 50, depth =4, eta =0.001	0.00	11989.94	0.18	0.58	11989.25	11990.56
Xgboost, nrounds = 50, depth =6, eta =0.001	0.00	11989.94	0.18	0.58	11989.25	11990.56
Xgboost, nrounds = 50, depth =8, eta =0.001	0.00	11989.94	0.18	0.58	11989.25	11990.56
Xgboost, nrounds = 50, depth =8, eta =0.01	0.00	4852.61	0.12	0.40	4852.14	4853.04
Random Forest, ntrees = 10	0.12	0.00	0.00	0.00	0.00	0.00
Xgboost, nrounds = 50, depth =4, eta =0.2	0.12	0.00	0.00	0.00	0.00	0.00
Xgboost, nrounds = 50, depth =4, eta =0.3	0.13	0.00	0.00	0.00	0.00	0.00
SuperLearner	NA	0.00	0.00	0.00	0.00	0.01

Table S7. Cross-Validated Coefficients and Risk for Total Deaths to-date

	COVID-19 Cases at Day 25				COVID-19 Cases Total to-date				Deaths at Day 100			Deaths Total to-date			
Cluster	Q1	Q2	Q3	Q4	Q1	Q2	Q3	Q4	Q1	Q2	Q3	Q1	Q2	Q3	Q4
1	9	16	67	292	5	21	64	294	40	66	278	17	24	64	279
2	213	323	320	220	202	278	364	232	532	336	208	288	265	288	235
3	193	166	129	85	192	186	141	54	356	141	76	234	158	108	73
4	254	171	104	58	265	163	84	75	454	91	42	346	124	73	44

Table S8. Breakdown of outcome quantiles across the county dendrogram clusters
